# NK cells from an AML patient have recovered in remission and reached comparable cytolytic activity to that of a healthy monozygotic twin mediated by the single-chain triplebody SPM-2

**DOI:** 10.1186/1479-5876-11-289

**Published:** 2013-11-16

**Authors:** Todd A Braciak, Sarah Wildenhain, Claudia C Roskopf, Ingo A Schubert, Georg H Fey, Uwe Jacob, Karl-Peter Hopfner, Fuat S Oduncu

**Affiliations:** 1 Division of Hematology and Oncology, Medizinische Klinik und Poliklinik IV, Klinikum der Universität München, Ziemssenstrasse 1, D-80336 Munich, Germany; 2 Department of Biochemistry and Gene Center, Ludwig-Maximilians-University, Munich, Germany; 3 Department of Biology, University of Erlangen-Nuremberg, Erlangen, Germany; 4 SpectraMab GmbH, Munich, Germany

**Keywords:** Single chain triplebody (triplebodies), Antibody-dependent cellular cytotoxicity (ADCC), Natural killer (NK) cells, Acute myeloid leukemia (AML), Cancer immunotherapy

## Abstract

**Background:**

The capacity of patient’s Natural Killer cells (NKs) to be activated for cytolysis is an important prerequisite for the success of antibody-derived agents such as single-chain triplebodies (triplebodies) in cancer therapy. NKs recovered from AML patients at diagnosis are often found to be reduced in peripheral blood titers and cytolytic activity. Here, we had the unique opportunity to compare blood titers and cytolytic function of NKs from an AML patient with those of a healthy monozygotic twin. The sibling’s NKs were compared with the patient’s drawn either at diagnosis or in remission after chemotherapy. The cytolytic activities of NKs from these different sources for the patient’s autologous AML blasts and other leukemic target cells in conjunction with triplebody SPM-2, targeting the surface antigens CD33 and CD123 on the AML cells, were compared.

**Methods:**

Patient NKs drawn at diagnosis were compared to NKs drawn in remission after chemotherapy and a sibling’s NKs, all prepared from PBMCs by immunomagnetic beads (MACS). Redirected lysis (RDL) assays using SPM-2 and antibody-dependent cellular cytotoxicity (ADCC) assays using the therapeutic antibody Rituximab^TM^ were performed with the enriched NKs. In addition, MACS-sorted NKs were analyzed for NK cell activating receptors (NCRs) by flow cytometry, and the release of TNF-alpha and IFN-gamma from blood samples of both siblings after the addition of the triplebody were measured in ELISA-assays.

**Results:**

Patient NKs isolated from peripheral blood drawn in remission produced comparable lysis as NKs from the healthy twin against the patient’s autologous bone marrow (BM) blasts, mediated by SPM-2. The NCR receptor expression profiles on NKs from patient and twin were similar, but NK cell titers in peripheral blood were lower for samples drawn at diagnosis than in remission.

**Conclusions:**

Peripheral blood NK titers and *ex vivo* cytolytic activities mediated by triplebody SPM-2 were comparable for cells drawn from an AML patient in remission and a healthy twin. If these results can be generalized, then NKs from AML patients in remission are sufficient in numbers and cytolytic activity to make triplebodies promising new agents for the treatment of AML.

## Background

Interest in the development of antibodies and antibody-derived agents such as antibody-drug conjugates (ADCs) and bispecific agents for the treatment of acute myeloid leukemia (AML) has increased over the past years. A prototypical agent, gemtuzumab ozogamycin (GO; Mylotarg^TM^), has been effective in the treatment of AML, but was withdrawn from the market because of side toxicities [1,2]. The problem causing this withdrawal was not related to the suitability of the myeloid surface antigen CD33 as a target, but rather to the chemical linker connecting the toxin component of this drug to the antibody carrier. CD33 remains a valuable and clinically validated target for the therapy of CD33-positive subtypes of AML with antibody-derived agents [3-5], and consequently, several major drug developers currently study new agents for the treatment of AML with specificity for CD33. One class of such agents relies on potent new toxins coupled with improved linkers to the antibody, such as the agent SGN-CD33a [6]. Another class recruits effector cells including NKs or T cells for the elimination of AML cells. This class includes the agent AMG-330 [7] recruiting T cells as effectors, as well as our team’s single chain triplebodies 123-16-33 [8] and 33-16-33 [9], recruiting NKs. It further includes CD33-specific antibodies with Fc domains engineered for improved binding to Fc-receptors on effector cells. Also, antibodies specific for CD123, the alpha subunit of the receptor for Interleukin-3, an important growth and differentiation factor for early hematopoietic cells and the myeloid lineage, have produced promising results in preclinical studies [10]. Antibodies against CD123 have mediated cytolysis of AML cells and in particular of AML-Leukemia Stem Cells (AML-LSCs) [11,12].

Our team has developed a new class of antibody-derived fusion proteins called triplebodies, suited for the elimination of AML cells [8,9,13-17]. These agents carry two binding sites for surface antigens on the cancer cell and one for a trigger molecule on an effector cell. They can bind two different tumor antigens on the same target cell and through this mode of “dual targeting” address the cancer cell with enhanced selectivity [15,16,18]. In addition, they bind a trigger molecule on an effector cell, such as CD16 on NKs, and thereby activate the effector for cytolytic elimination of the cancer cell. Triplebody SPM-2 employed in the present study carries single chain Fv (scFv) antibody fragments specific for CD33 and CD123 on AML cells linked in a single polypeptide chain to an scFv module specific for CD16, the low affinity Fc gamma RIII receptor on NKs and macrophages and a few other cells of the hematopoietic system [8]. SPM-2 was designed for the elimination of both bulk AML cells and AML-LSCs, because both are double-positive for this pair of antigens.

LSCs from patients with most subtypes of AML however carry a higher combined cell surface density of this pair of antigens than bulk AML cells, normal hematopoietic cells, and healthy hematopoietic stem cells (HSCs) [3,11,19,20]. SPM-2 was therefore designed for a preferential elimination of AML-LSCs *in vivo*. It is still unknown whether this objective can be reached because the agent has still not been tested in humans. It is important to eliminate not only bulk AML cells, but also the LSCs, because the LSCs are believed to be the relevant subset within the population of minimal residual disease (MRD) cells, which are responsible for relapse and poor disease outcome [4,21-23]. New agents intended to induce a deeper remission and to prolong the time to relapse must therefore make a deliberate effort to target the MRD cells. Currently available chemotherapeutic agents achieving remission for many AML patients probably also act (at least in part) through the elimination of some LSCs. However, most often they do not achieve long-lasting complete remissions and have not been designed with the intent to specifically eliminate MRD cells but rather bulk AML cells. A well-defined immunophenotype of MRD cells is not available, which would lend itself as a specific address for the design of antibody-derived agents. However, in a retrospective clinical study the MRD cell compartment responsible for early relapse was tentatively equated with the CD34^+^ CD38^-^ CD123^+^ subset of AML cells [24]. If these results can be confirmed by prospective studies, then triplebody SPM-2 could be especially promising for the therapeutic removal of AML-MRD cells. Its ability to reach this objective critically hinges upon the availability of sufficient numbers of active NKs in AML patients at the time of the intended use of the agent.

The compartment of functional NKs is often reduced in AML patients [25-29]. This impairment has been attributed to different causes, among them a down-regulated cell surface expression of the activating natural cytotoxicity receptors (NCRs) NKp30, NKp44 and NKp46 [25-27,29,30]. Low-level expression of NCRs (NCR^dim^) on patient-derived NKs was correlated with poor prognosis in AML, as patients with NCR^dim^ NKs had significantly lower 5-year survival rates than matched patients with NCR^bright^ NKs [26]. Deficiencies in cytokine release have also been linked to an impairment of NK cell function in AML patients. The capacity of NKs to secrete IFN-gamma was highly impaired in AML patients and was correlated with suppressed immune responses against autologous leukemic cells [31-33]. Finally, in adult acute leukemia, impaired production of cytokines by NKs was associated with early relapse [34].

Triplebody SPM-2 was not designed as a frontline therapeutic for the initial debulking of AML blasts. This objective is reached in most cases by the initial chemotherapy. Rather, SPM-2 was designed to be used after chemotherapy when the blast titer is greatly reduced for most patients and when the titer of functional NKs is expected to have at least partially recovered towards normal levels [28]. Therefore, here we studied the effectiveness of SPM-2 to mediate lysis of an AML patient’s BM blasts as targets, in combination with autologous NKs drawn from peripheral blood at diagnosis and in remission. The analysis was performed in comparison with NKs isolated from the patient’s healthy monozygotic twin. This exceptional situation provided us with a unique opportunity to compare the activity of the patient’s NKs with NKs genetically as closely identical as possible from a healthy donor.

Here the patients NKs, drawn in remission, were restored to titers in the blood comparable to those of the healthy twin and were able to achieve effective lysis of autologous AML blasts in conjunction with SPM-2. Notably, they also achieved comparably effective lysis of an established lymphoma-derived target cell line mediated by the reference antibody Rituximab^TM^, and had similar expression profiles of NCRs as the healthy sibling’s cells. The present study provides a precedent case in support of our hypothesis that SPM-2, administered at appropriate stages in the course of a standard therapy for AML, will become useful as an adjuvant drug.

## Methods

### Expression in mammalian cells, purification and protein-chemical characterization of triplebody SPM-2

The DNA construct coding for SPM-2 was based on the published triplebody 123-16-33 [8] and was synthesized by a commercial provider (Eurofins/MWG-Operon, Ebersberg; Germany). The CD16-specific scFv was stabilized following published procedures [8,35] and humanized. Humanization of the CD123- and CD33-specific scFvs and the stabilization of the CD123-specific scFv followed similar procedures (S. Wildenhain, I. Schubert, A. Honegger et. al., unpublished data). The N- and C-terminal CD33- and CD123-specific scFvs were separated from the central CD16-specific scFv by (G_4_S)x4 linkers. For expression purposes, human 293 F cells (DSMZ; German Collection of Microorganisms and Cell Lines; Braunschweig, Germany) were stably transfected with plasmid DNA using the 293fectin^TM^ transfection reagent (Life Technologies, Darmstadt, Germany) according to the manufacturer’s instructions in a total volume of 30 ml. The cells were then cultured under continuous selection with hygromycin C. The recombinant protein was captured from culture supernatants via its C-terminal hexahistidine tag by retention on a metal-ion affinity matrix and purified by anion- and cation-exchange chromatography. Concentrations of the purified protein were determined by spectrophotometry and calculated using the molar extinction coefficient derived from the amino acid sequence. The resulting final protein meets current regulatory standards and industry norms, and was named SPM-2 to indicate its status as a candidate for clinical development. Equilibrium binding constants (K_D_) of each of the individual scFvs of SPM-2 were in the 20–30 nM range, and thus were similar to the values reported for the initial agent [8].

### Preparation of primary cells from blood and bone marrow of human donors

Peripheral blood and bone marrow samples were drawn from subjects into EDTA solution after receiving informed consent. The project was approved by the Ethics Committee of the University of Munich Medical Center. Bone marrow mononuclear cells (BMMC) containing the leukemic blasts and Peripheral Blood Mononuclear Cells (PBMCs) were enriched by ficoll density centrifugation using the Lymphoflot reagent (Biotest, Dreieich, Germany) according to manufacturer’s instructions. Isolated cells were then either suspended in RPMI medium (Life Technologies) containing 10% fetal bovine serum (FBS) with penicillin and streptomycin (PS) at 100 units/ml and 100 μg/ml, respectively, for immediate use, or stored frozen in a solution containing 90% FBS and 10% DMSO for future use. Cell viability was assessed by trypan blue exclusion.

### Ex vivo expansion of MNCs from healthy donors in the presence of IL-2

PBMCs were expanded *ex vivo* in RPMI medium containing Interleukin-2 (IL-2) plus 5% human serum (Life Technologies) for 20 days as described [16,36], and were then frozen in aliquots for subsequent use. Prior to use in cytolysis experiments, the cells were thawed and cultured overnight in RPMI medium containing 5% human serum plus 50 units/ml and 50 μg/ml PS, respectively, but no additional IL-2**.**

### Flow cytometric analysis

Flow cytometric analysis was performed with an Accuri C6 flow cytometer (BD Biosciences, Heidelberg, Germany). The CD3-, CD16-, and CD56-specific monoclonal antibodies (mAbs) used for the analysis of NK cell content in PBMC-preparations as well as isotype control mAbs were from Immunotech (Marseille, France), while the NKp30-, NKp44-, NKp46-specific and isotype control mAbs used for the analysis of NK cell receptors (NCRs) [37] on isolated NKs were from eBioscience (Frankfurt, Germany). Cell surface densities of CD33 and CD123 were measured using a calibrated cytofluorimetric assay as described [8,35]. For this purpose, a commercial kit of fluorescent beads with known numbers of fluorescent chromophores per bead (QIFIKIT®; DAKO; Hamburg, Germany) was employed, as well as fluorescent-labeled mAbs. This procedure allows the investigator to express the measured fluorescent intensity of mAbs bound to the surface of the patient’s cells in terms of average number of antigen copies per cell [38].

### Antibody Dependent Cellular Cytotoxicity (ADCC) and Redirected Lysis (RDL) assays using Calcein release

In this study we refer to cell-mediated cytolysis assays with whole antibodies as “ADCC” and to tests with antibody-derived agents such as triplebodies as “redirected lysis (RDL)” assays. Non-radioactive cytolysis assays based on the release of calcein from target cells pre-labeled with calcein AM (Life Technologies) were performed as described [16,39]. The cytolytic activity of NKs from various sources was calibrated in standard ADCC assays with the commercial CD20-mAb Rituximab^TM^[39,40] as the mediator of lysis and lymphoma-derived Raji cells [41] as targets. This calibration allowed us to assess the cytolytic activity of NKs from various sources using a standard mAb and a commonly used target cell line, and thus to make our results comparable to the current benchmarks in the field. For the calibration reaction with Rituximab^TM^, untouched NKs were first enriched by the MACS kit (Miltenyi kit; see above) from PBMC samples and then used at a constant effector-to-target (E : T) ratio of 2.5 : 1 against Raji targets. The same NKs were also used in redirected lysis experiments with the patient’s autologous bone marrow AML blasts as targets in conjunction with SPM-2. Specific lysis was measured by quantitating the release of calcein from target cells using a fluorimeter/ELISA plate reader and expressed in relative light units (RLU) at 485/535 nm. Calcein release was measured at 3 and 4 hour time points for ADCC and RDL experiments, respectively. Specific cellular cytotoxicity was expressed as overall lysis minus the background of spontaneous lysis mediated by the NKs alone, in the absence of added antibody-reagents. Specific lysis was evaluated by the formula:

% specific lysis = 100 * (Experimental Release RLU – Background Release RLU)/(Maximal Release RLU – Background RLU).

### Enrichment of human NK cells by preparative sorting with immunomagnetic beads

NKs were enriched by negative selection using a commercial NK cell isolation kit (Miltenyi Biotec MACS sorting kit, Bergisch Gladbach, Germany) according to manufacturer’s instructions. The enriched cells are referred to as “untouched” NKs, because as a result of the negative selection, no residual mAb is bound to their surface and they have not been eluted from an affinity matrix with harsh reagents. Starting material were ficoll-hypaque purified PBMCs from blood samples of the donors. The enriched NKs were then either resuspended in RPMI medium containing 10% FBS with PS at 100 units/ml and 100 μg/ml, respectively, for use in ADCC and RDL assays, or placed in PBS solution containing 1% bovine serum albumin (BSA) for flow cytometric analysis.

### Measurement of IFN-gamma and TNF-alpha release into peripheral blood samples by ELISA assays

Concentrations of human cytokines IFN-gamma and TNF-alpha were measured with ELISA kits (eBioscience) following manufacturer’s instructions. Triplebody SPM-2 was added to peripheral blood samples at concentrations of 10, 1, or 0.1 nM , and the reaction mixtures were then cultured for 6 h at 37°C in 96 well round bottom Nunc plates in 200 ul volumes. Blood samples were frozen and stored at -20°C and were thawed only immediately before use in cytokine assays. In addition, cytokine release was studied with the same ELISA kits in supernatants from the 4 h RDL and 3 h ADCC assays, in which MACS-purified NKs had been used as effectors against autologous BM blasts as targets.

### Statistics

All statistical analysis was performed by STATVIEW 4.5 programs from Abacus Concepts (Berkeley, CA) using Student’s *t*-test for the final determination of significance (p < 0.05).

## Results

### SPM-2 mediates efficient lysis of BM leukemic blasts from a patient with FAB M1 AML in conjunction with effector cells from a healthy unrelated donor

**Figure 1:**
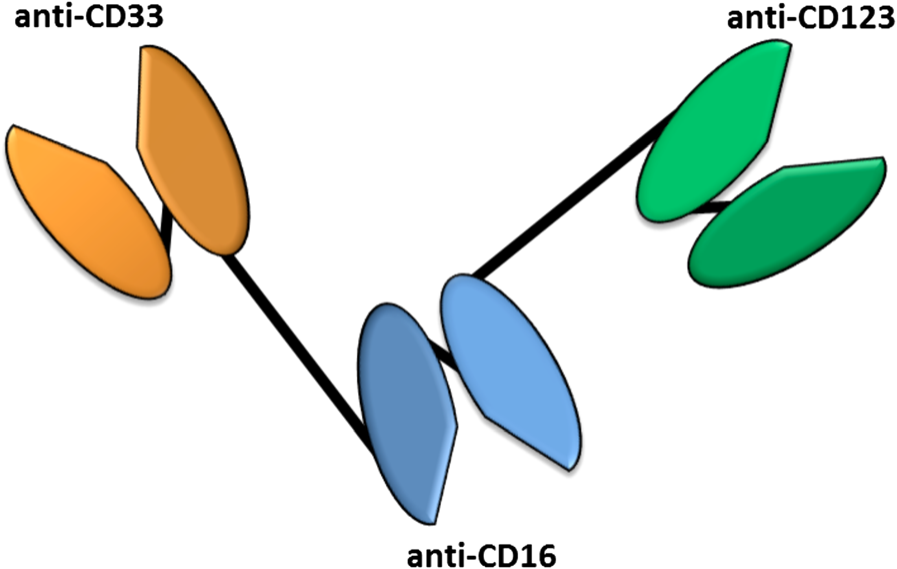
**Structure of single chain triplebody SPM-2.** ScFv domains (colored in the diagram) specific for CD33, CD16 and CD123, respectively, were connected by two (Gly_4_Ser)x4 linkers (black lines) into a single polypeptide chain. The distal scFv domains specific for CD33 (orange) and CD123 (green), bind targets on the same AML blast while the central scFv domain (blue) binds CD16 on NK cells. Antigen binding sites are indicated by the triangular pockets.

To evaluate the ability of SPM-2 (Figure 1) to mediate lysis of blasts from a patient diagnosed with an AML FAB M1, RDL experiments were performed with isolated BMMCs obtained from the patient at diagnosis as targets. The BMMCs prepared in this manner contained 89.7% AML blasts, as determined by morphology and cytological criteria (Table 1). Five hundred million (5 x 10^8^) MNCs were recovered from a 5 ml BM sample of this patient, drawn at diagnosis, and some aliquots were cryopreserved for further analysis.

**Table 1:** Data and immunophenotype of AML FAB-M1 patient BMMCs

**Gender**		**Female**	
Age:		21 years old	
Diagnosed with:		AML FAB-M1; AML without maturation according to WHO classification [42]	
Blast titer:		89.7% blast in the marrow at diagnosis	
Case history		One course of induction chemotherapy following HAM protocol high dose cytarabine with mitoxantrone [49]	
Current status:		Complete remission acheived by induction chemotherapy (HAM)	
Myeloid cells % (in blast gate)		Co-expression (cross-lineage) % (in blast gate)	
CD 34	89.6%	CD34/CD56	47.9%
CD 33	<19%	CD34/CD7	12.3%
CD 123	n.d. at diagnosis	CD34/HLA-DR	90.5%
CD 117	< 1%	CD34/CD7	12.3%
CD 38	89.9%		
cy MPO	41.7		

n.d. not determined.

Cy MPO cytoplasmic myeloperoxidase; flourescent marker enzyme for the myeloid lineage.

**Figure 2:**
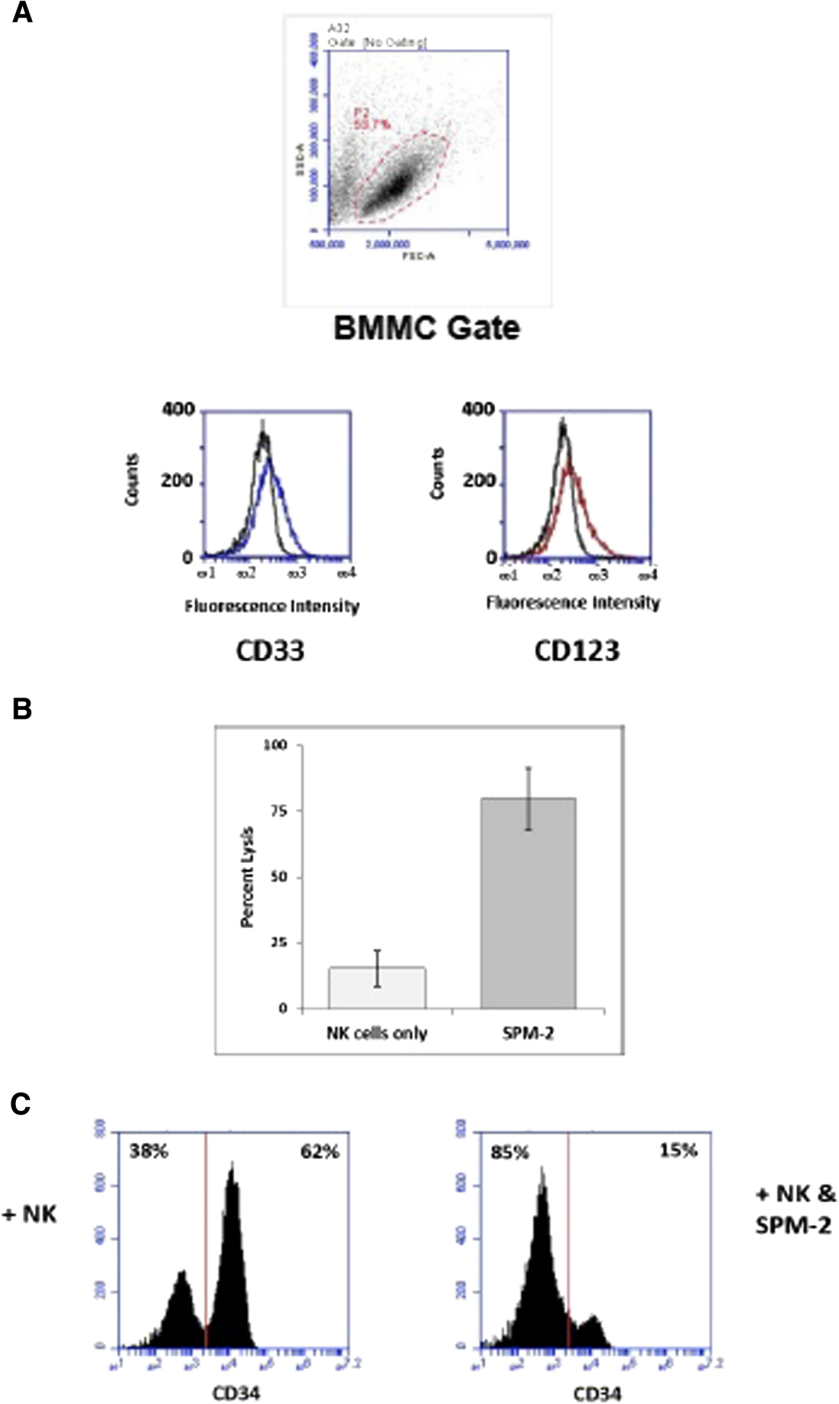
**BM blasts from an FAB M1 AML patient are lysed efficiently in a RDL reaction by effectors from an unrelated donor, mediated by SPM-2. (A)** Patient isolated BMMC were stained with fluorescent antibodies specific for CD33 and CD123, respectively, in comparison to isotype controls. P2: gate used for analysis of live BMMCs allowing their discrimination from dead cells. Mean fluorescence Intensities measured for each antigen were converted to antigen densities by calibrating fluorescence intensities with the QuiFi kit (Methods). **(B)** MNCs from an unrelated healthy donor, expanded *ex vivo* in the presence of IL-2 (Methods), were used as effectors at an MNC : target (E:T) ratio of 10 : 1 in a 4 h RDL assay. This corresponds to an E:T ratio of NK cells : targets of 2.5 : 1, because NK cells were 25% of all cells in this expanded MNC population. Targets were BMMCs from the AML patient obtained at diagnosis, pre-labeled with calcein AM. SPM-2 was added at 10 nM concentration (right bar). The control reaction was allowed to proceed without added agents (left bar; NKs alone). Cellular lysis was measured by calcein release and plotted as fraction (%) of maximum release. Error bars: arithmetic means over 3 separate experiments (n = 3). **(C)** Flow cytometric analysis after an RDL reaction. Patient’s BMMCs were treated with effector cells and SPM-2 in a 4 h RDL experiment as in panel A. At the end of the reaction, cells were stained with a fluorescent-labeled CD34-specific mAb and analyzed by FACS. Numbers of CD34^pos^ cells are plotted against the intensity of CD34-specific fluorescence. Left panel: after reaction with effectors alone; right panel: after reaction with effectors plus SPM-2. Numbers in upper right corner: percent of CD34^pos^ cells surviving at the end of the reaction.

Among the isolated BMMCs, 89.6% expressed detectable levels of CD34, consistent with the diagnosis of a FAB M1 AML. The FAB M1 AML subtype commonly displays very immature blasts and a high percentage of CD34-positive cells. It is called “AML without maturation” in the WHO classification [42] and often leads to low white blood cell counts. At diagnosis less than 1% of the BMMCs expressed cell surface CD33, leading to the initial diagnosis of a “CD33-negative AML”. Expression data for CD123 at diagnosis are not available. Subsequent cytofluorimetric analysis gated on the BMMC population (consisting of approx. 90% AML blasts) performed under more sensitive conditions (calibrated cytofluorimetry; Figure 2A) revealed a mean expression of 130 and 230 copies per cell of CD33 and CD123, respectively, in this population of cells. These are very low levels, considering that blasts from other AML subtypes can commonly carry up to several thousand and sometimes several ten-thousand copies per cell of these antigens [8,43,44].

This patient’s AML blasts were lysed efficiently by SPM-2 in combination with MNCs expanded *ex vivo* for 20 days with IL-2 from a healthy unrelated donor used as a source of effectors (Figure 2B). Net lysis of the targets by the effector cells alone was 15 ± 11%, net lysis in the presence of a saturating (10 nM) dose of SPM-2 plus effectors was 80 ± 20% (Figure 2B). Thus, specific lysis induced by the triplebody was approximately 65%. This is a surprisingly high value for a 4 h assay, considering the low surface density of both target antigens.

The majority of AML-LSCs are contained in the CD34-positive subset of bone marrow cells [11,45]. Therefore we analyzed, whether SPM-2 was capable of mediating lysis of this subset, as a first step towards answering the key question, whether SPM-2 can mediate lysis of AML-LSCs. As a result, in a 4 h RDL-reaction using isolated AML BMMCs as targets and *ex vivo* expanded MNCs from an unrelated donor as effectors, SPM-2 mediated a significant reduction of the CD34-expressing subset to 15%, down from 89.6% at the start of the reaction (Figure 2C, right panel). Exposure to the MNC effectors alone reduced the CD34-positive blasts down to only 62% (Figure 2C, left panel). Thus, the net effect of treatment with SPM-2 was a specific depletion of the CD34-bearing cells by 47%.

Beyond the CD34-positive compartment, the majority of AML-LSCs are typically found in the CD34^pos^ CD38^neg^ subset, although some LSCs are also present in other cellular subsets [19]. Moreover, the AML MRD cells relevant for relapsed disease have been reported to be predominantly contained in the CD34^pos^ CD38^neg^ CD123^pos^ compartment [24]. Therefore, in an attempt to determine, whether SPM-2 was capable of eliminating AML-MRD cells in a narrower cellular subset beyond the broader CD34^pos^ compartment, we analyzed whether SPM-2 also affected the CD34^pos^ CD38^neg^ CD123^pos^ subset within the AML BMMC sample in combination with IL-2 expanded effector cells from a healthy donor. The experiment was performed using a similar 4-color flow cytometric analysis as reported by others [24].

As an initial result, obtained with limited numbers of cells, depletion of the CD34^pos^ CD38^neg^ CD123^pos^ subset was observed following treatment with SPM-2 plus allogeneic effectors in comparison to treatment with MNCs alone (data not shown). For this patient, the CD34^pos^ CD38^neg^ cells represented approximately 1.4% of the total BMMCs. This value falls into the typical range reported for this subset by other authors [24]. Taken together, this set of data provides a first indication in favor of our contention that SPM-2 is capable of eliminating cellular subsets enriched in AML-LSCs and MRD cells through the recruitment of activated NKs. While this result is consistent with the claim that SPM-2 can eliminate LSCs, definitive proof requires further and more stringent analysis than we were able to perform here due to the scarcity of this BMMC sample.

### Cytolytic activity of patient’s NKs drawn at diagnosis and in remission compared with NKs from a healthy monozygotic twin

Next, we wished to determine whether SPM-2 was capable of mediating lysis of the patient’s AML blasts by autologous NKs. For this patient, we had the rare opportunity to obtain NKs from peripheral blood both at diagnosis and in remission after completion of the induction chemotherapy, and to compare their titers and cytolytic activities to NKs isolated from a healthy monozygotic twin.

**Figure 3:**
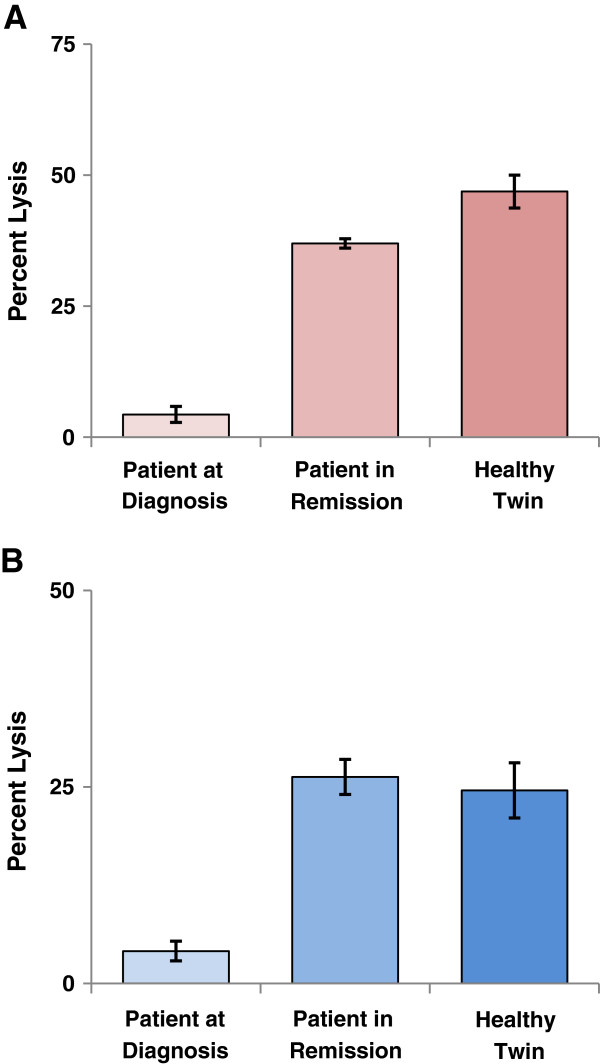
**NK cells from PBMCs of the patient in remission and the healthy twin have comparable cytolytic activity. (A)** MACS sorted NKs isolated from PBMCs of the patient drawn at diagnosis (left bar), in remission (central bar) and from the healthy twin (right bar) were used in RDL assays against calcein labeled BMMCs from the patient at diagnosis as targets (red). 4 h RDL reactions were run at an E : T ratio of 2.5 : 1 and incubated with a 10 nM dose of SPM-2 versus NKs alone as control. Error bars: arithmetic means over 3 separate experiments (n = 3). **(B)** The same NK effector populations as in panel **A** were also used in 3 h ADCC reactions at the same E : T ratios. Target cells were calcein labeled Raji cells (CD20^pos^). Reactions mediated by 10 μg/ml (approx. 70 nM) of the commercial CD20-antibody Rituximab^TM^ (blue). Error bars: arithmetic means over 3 separate experiments (n = 3).

As a result, MACS-purified NKs drawn from the sibling and the patient in remission showed similar cytolytic activity mediated by SPM-2 towards the patient’s AML blasts (Figure 3A, red). Mean specific lysis obtained with NKs from the sibling was 47 ± 7% versus 37 ± 2% for NKs from the patient in remission. These values are arithmetic means over 3 independent experiments (n = 3). By contrast, the cytolytic activity of the NKs drawn from the patient at diagnosis was significantly lower. With equal numbers of effector cells used as an input for each reaction, only 4.3 ± 2.6% specific lysis was observed with a saturating dose of SPM-2 (10 nM; Figure 3A, left bar).

Finally, we wished to determine whether these comparable activities of the patient’s and sibling’s NKs were only observed with the patient’s AML blasts as targets, or whether this was an intrinsic property of these NKs, also observed for other targets. Therefore, a benchmark ADCC experiment was performed with CD20-positive Raji lymphoma-derived cells as targets (Figure 3B, blue), the commercial CD20 antibody Rituximab^TM^ as the therapeutic agent, and with immunomagnetically purified NKs as effectors. As a result, comparable lytic activity was found again for NKs from the patient in remission and the twin (Figure 3B). Here, NKs from the patient in remission produced 26 ± 4% specific lysis using a standard dose of Rituximab^TM^ compared to 25 ± 8% for the sibling’s NKs. NKs from the patient at diagnosis also produced lower specific lysis in this assay (4 ± 2%).

Taken together, these results indicate that the peripheral blood NKs from the patient in remission are comparable in cytolytic activity to those of the healthy twin in response to SPM-2. One needs to bear in mind, however, that this patient was younger than the average AML patient at diagnosis and that response to therapy is usually better for younger patients.

### Titers of AML blasts and NK cells in peripheral blood at diagnosis and in remission

**Figure 4:**
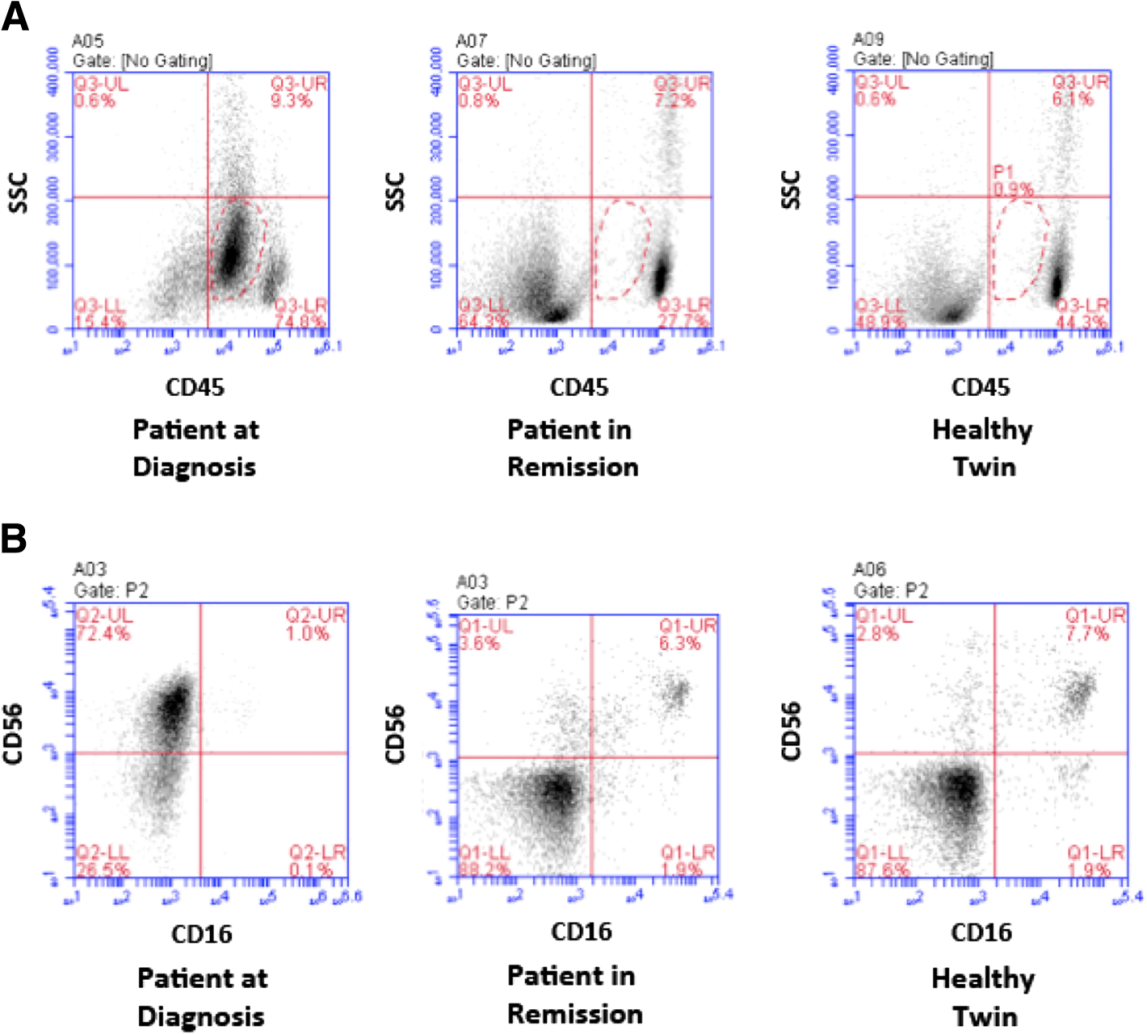
**Analysis of AML blasts and NKs within PBMCs from the different donor samples. (A)** Blast content among PBMCs from the patient at diagnosis and in remission (left and central panels) or from the healthy twin (right) is shown. PBMCs were stained with a FITC-labeled CD45 mAb and analyzed against side scatter (SSC) by flow cytometry. CD45^dim^ x SSC^LOW^ cells have been defined as AML blasts [24]. Dashed line: P1 gate (blasts). Numbers in the corners of quadrants: fraction of total cells falling into this quadrant. **(B)** Analysis of NK cell content. PBMCs form the same sources as in A were stained with FITC-labeled CD16 and PE-labeled CD56 mAbs. NKs were defined as CD16^pos^CD56^pos^ cells (upper right quadrant). The population in the upper left corner in the left panel is the unusual population of CD56^bright^CD16^neg^ cells, previously reported in AML samples [30] as an NK subset that exclusively produces cytokines.

To look for explanations for the apparent deficiency in cytolytic activity of the NKs from the patient at diagnosis, frequencies of NKs and leukemic blasts in the various PBMC samples were assessed by flow cytometry (Figure 4). Plots of side scatter (SSC) vs. fluorescence intensity of cells stained with a FITC-labeled CD45 mAb revealed a well-defined population of CD45-FITC^dim^ x SSC^low^ cells (P1 in Figure 4A). These cells have been identified as AML blasts by other authors [24,46]. Among the patient’s PBMCs drawn at diagnosis, this subset was abundant (58%; Figure 4A, left panel), while it accounted for only about 1% of all PBMCs from the patient in remission and the healthy twin (Figure 4A, central and right panels).

NKs were identified in cytofluorimetric studies as CD16^pos^CD56^pos^ cells (Figure 4B). Only 1% of the patient’s PBMCs at diagnosis fell into this gate (Figure 4B, left panel), whereas 6.3 and 7.7% of all PBMCs had this phenotype for the patient in remission and the healthy sibling (Figure 4B, central and right panels). This finding supports our initial expectation that for an AML patient in remission the NK cell compartment should be restored to almost normal levels. It also is the most likely explanation for the results shown in Figure 3, where we observed that NKs from the patient at diagnosis had lower cytolytic activity in ADCC and RDL assays than the corresponding cells from the patient in remission and her healthy twin. These data suggest that the deficiency in cytolytic function observed at diagnosis most likely was due to reduced NK cell titers.

Interestingly, a high percentage of CD56^bright^ CD16^low^ cells were found in PBMCs of the patient at diagnosis (Figure 4B, left panel, upper left quadrant). This population has previously been reported by other authors to be increased in AML patients and has been described as a characteristic NK cell subset with elevated production of cytokines [30]. This result is consistent with the diagnosis of this patient showing an unusually high percentage of CD34^pos^CD56^pos^ cross-lineage cells amongst the AML blasts (47.9% of blasts; Table 1).

### Enrichment of NK cells by MACS sorting and titers of T cells in PBMCs

**Figure 5:**
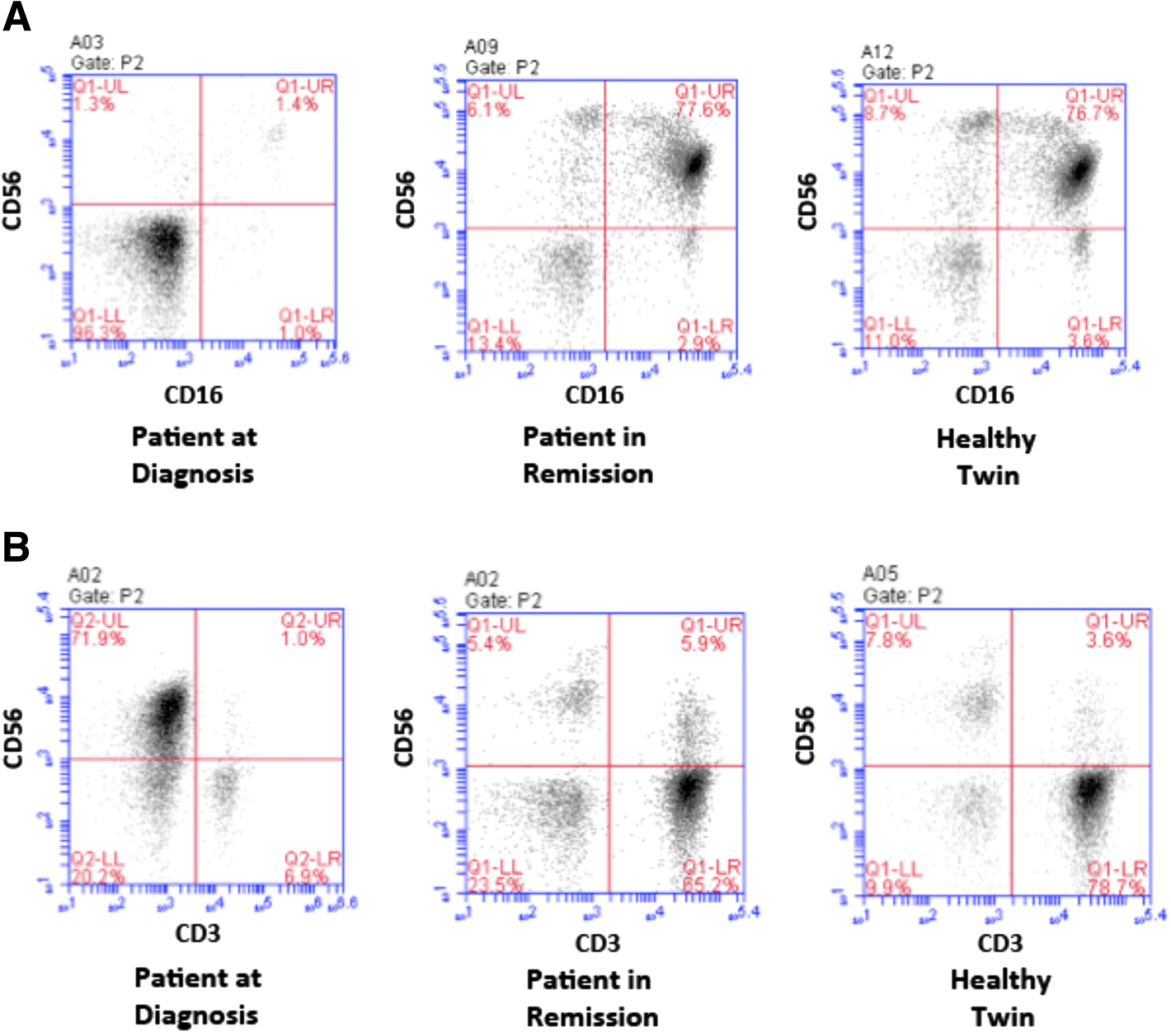
**Analysis of MACS sorted NKs and T cells within PBMCs isolated from the different donor samples. (A)** The CD16^pos^CD56^pos^ NK cells enriched by immunomagnetic beads (MACS; Methods) are shown in the upper right quadrant. Events in the lower left quadrant in the sample from the patient at diagnosis are considered to be very immature blasts devoid of lineage markers as discussed in the text. **(B)** CD3^pos^ CD56^neg^ cells (lower right quadrant): T cells.

After preparative enrichment by MACS sorting, CD56^bright^CD16^bright^ NKs accounted still for only 1.4% of total cells in the sample from the patient at diagnosis (Figure 5A, left). This poor enrichment probably reflects the high content of AML blasts with very immature, early progenitor phenotype, devoid of lineage markers, in the PBMC sample taken at diagnosis. Therefore, negative sorting using antibodies specific for lineage markers did not remove these cells. In contrast, the MACS-enriched CD56^bright^CD16^bright^ NKs accounted for approx. 77% of all cells in the corresponding samples from the patient in remission and the healthy twin used in the RDL and ADCC assays (Figure 5A; central and right panels). These observations are consistent with previously reported decreased frequencies of NKs in the blood of AML patients [30].

Finally, an approximate 10-fold reduction of the T cell compartment was also observed comparing PBMCs from this patient drawn at diagnosis with those obtained in remission and those from the healthy twin (Figure 5B). Only 6.9% of the patient’s PBMCs drawn at diagnosis were CD56^neg^CD3^pos^ T cells, compared with 65.2% and 78.7%, respectively, for the cells from the patient in remission and the healthy twin. This finding indicates that in the remission stage after the initial chemotherapy, not only NKs are restored but also the T cell compartment recovered to approximately normal levels.

### Expression levels of natural cytotoxicity receptors (NCRs) in PBMC samples

**Figure 6:**
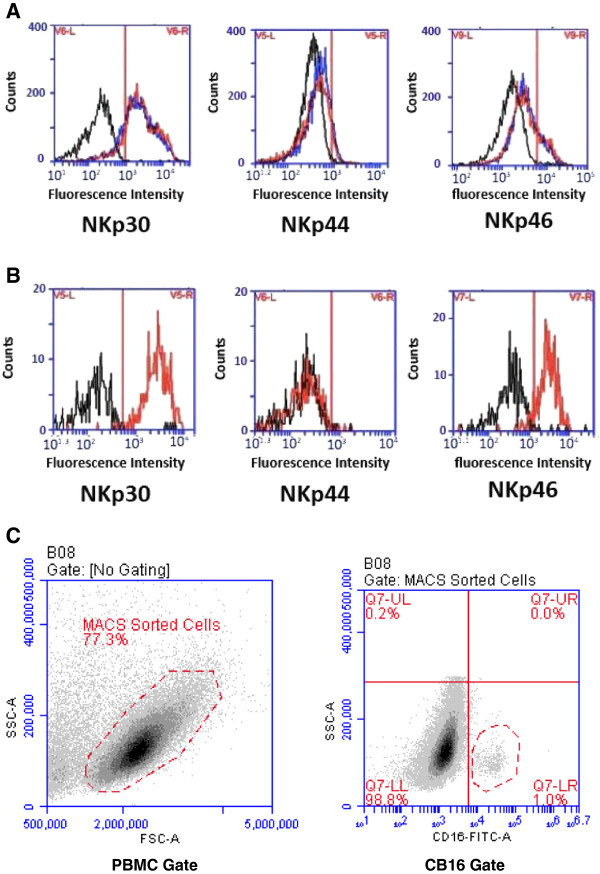
**Unaltered expression patterns of the NCRs NKp30, NKp44 and NKp46 on enriched NKs from the patient at diagnosis, in remission and the healthy twin. (A)** Expression pattern of NCRs on enriched NKs from the patient in remission (red) and the healthy twin (blue) compared with cells stained with an isotype control mAb (black). **(B)** To analyze NCR expression on enriched NKs from the patient at diagnosis, an additional sorting step for CD16 positive cells was performed to focus the analysis on the small subpopulation of NKs. NKs from the patient at diagnosis (red) are compared with cells stained with an isotype control mAb (black). **(C)** Gates chosen to generate data shown in panels **A)** and **B)**. Left: gate chosen to identify MACS-sorted cells (negative selection for NK cells). Right: additional gate used for identification of the CD16 pos subset (lower right quadrant) of the MACS sorted cells from the patient sample obtained at diagnosis (Methods).

As impaired cytolytic activity of NKs from AML patients had been proposed to be caused by reduced expression of NCRs [25,26], expression of NKp30, NKp44 and NKp46 on enriched NKs was studied (Figure 6). No significant difference in the expression patterns was observed between MACS enriched NKs from the patient in remission versus the healthy sibling (Figure 6A). The corresponding analysis for NKs from the patient at diagnosis was rendered more difficult by the fact that NKs were of low abundance in the MACS-enriched sample at this time. This analysis was therefore refined by further gating for CD16^pos^ cells and then analyzing the NCRs on this gated population of NKs. In this manner, an essentially unchanged NCR expression profile was found for these gated NKs (Figure 6B). The gating strategy used for this analysis of the PBMC sample drawn at diagnosis is shown in Figure 6C. From these combined observations, we conclude that for this particular patient the NCR expression profiles remained unchanged and that changes in the levels of NCRs are unlikely to be the cause of the impaired cytolytic functions recorded here.

### Whole blood samples from the AML-patient and the healthy sibling show different levels of cytokine release after addition of SPM-2

**Figure 7:**
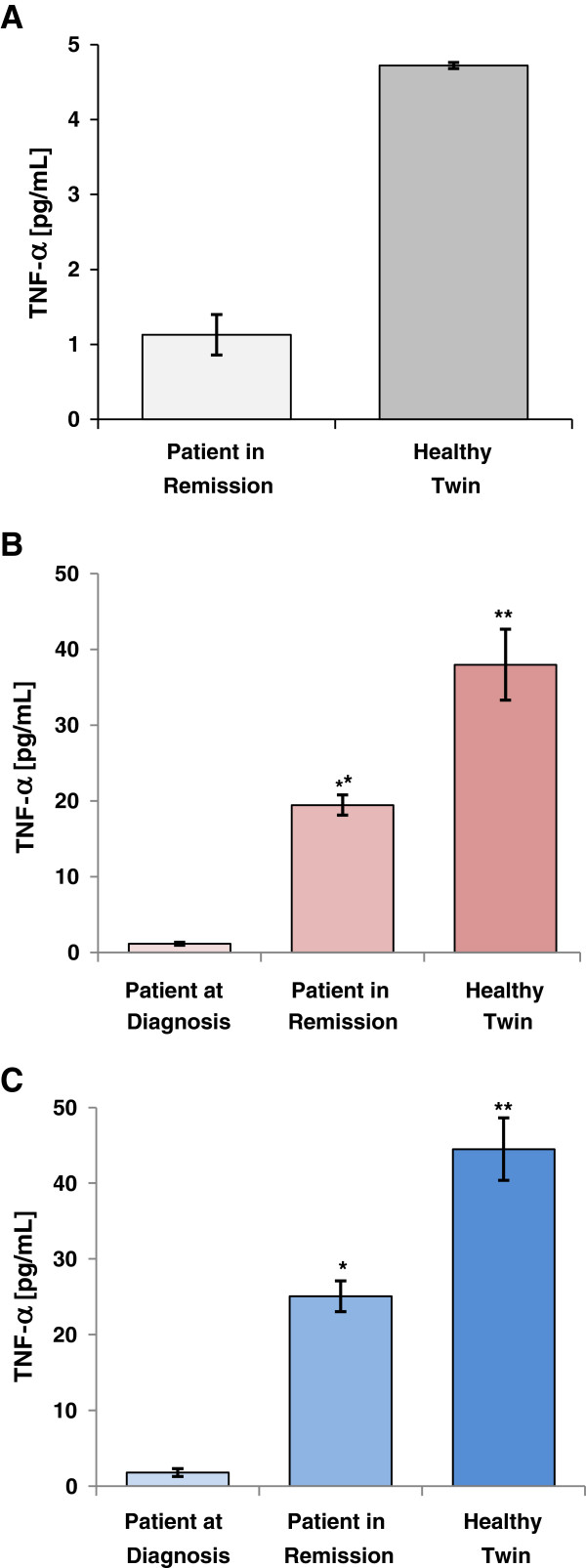
**Secretion of TNF-alpha from whole blood or from supernatants of cytolysis reactions after treatment with SPM-2. (A)** Release of TNF-alpha into whole EDTA treated blood from the patient in remission versus the healthy twin after addition of SPM-2. 200 μl samples of EDTA-blood were incubated for 6 h at 37°C with a 10 nM dose of SPM-2. Secretion of TNF-alpha was measured with a commercial ELISA kit (Methods). **(B)** Supernatants from a 4 h RDL assay using 10 nM dose of SPM-2 and the patient’s autologous BMMCs drawn at diagnosis as targets (red). Effector cells were MACs enriched NKs isolated from PBMCs taken from the patient at diagnosis (left), in remission (center), or from the healthy sibling (right). Secreted TNF-alpha was measured as in panel **A**. Plotted data are the arithmetic means over 6 independent experiments (n = 6). Error bars represent the standard error of the mean (SEM). Statistical significance was reached with p = .002 (*) and .004 (**) for the differences between cells from the healthy twin and the patient in remission versus at diagnosis, respectively. **(C)** Supernatants from a 3 h ADCC assay using 10 μg/ml dose of Rituximab and Raji leukemia cells as targets were assayed for TNF-alpha secretion using the same NK effector populations as for panel **A** (blue). Plotted data are mean values over six independent experiments (n = 6). Statistical significance was reached with p = .0001 (*) and .0003 (**), for the differences between healthy twin and patient in remission versus at diagnosis, respectively.

Reduced cytokine (IFN-gamma) release by NKs from AML patients had been reported to be correlated with suppressed immune responses against autologous leukemic cells [32,33,45]. Therefore, we tested whether differences in cytokine release were detectable after addition of SPM-2 either to whole blood samples from the patient drawn at diagnosis or those from the patient in remission and the twin. Determinations of IFN-gamma release by ELISA produced no signal above the detection threshold (4 pg/ml). However, TNF-alpha was detected in the corresponding samples after addition of SPM-2 (Figure 7A). Following incubation for 6 h with a saturating dose of SPM-2 (10 nM), 4.7 ± 0.1 pg/ml of TNF-alpha were detected in blood from the healthy sibling versus only 1.1 ± 0.1 pg/ml in the patient’s sample drawn in remission (Figure 7A). Thus, whole blood cells from the patient in remission showed a reduced release of TNF-alpha in response to the addition of SPM-2, compared to cells from the healthy sibling. This differential cytokine response does not appear to have affected the cytolytic activity mediated by SPM-2, as these two sources of NKs did not show major differences in our RDL and ADCC assays with autologous and allogeneic leukemic cells as targets.

To investigate, whether cytokine release during cytolytic reactions with MACS-purified NKs from these different sources was similarly independent of target antigen and the type of target cell line, we analyzed the cytokine release occurring during the ADCC and RDL reactions, by assaying the supernatants of these reactions in our ELISA assay (Figure 7B and C). TNF-alpha production was reduced for the reaction with NKs from the patient in remission relative to the healthy sibling but even more so with NKs from the patient at diagnosis. This result was observed for the supernatants from both the ADCC and RDL assays. IFN-gamma release was undetectable. These results are consistent with previous reports indicating that NKs from AML patients are attenuated in their release of cytokines [27,29].

## Discussion

The most important result of this study is that it helped to dispel concerns raised by critics about the use of triplebodies for the treatment of AML, because they rely on NK cells as effectors. The main argument was that too few NK cells were present in an AML patient for triplebodies to be effective. The critics held that it would be preferable to recruit T cells rather than NK cells as effectors, because T cells were present in greater numbers, because they have higher intrinsic cytolytic activity, are capable of serial lysis of several target cells in a consecutive manner, and can be stimulated to proliferate as a result of exposure to the agent. We cannot address all of these points here in detail, and some of our arguments regarding the relative merits of recruiting NK cells vs. T cells have been reviewed elsewhere [17]. Suffice it to state that NKs are also capable of serial lysis and that their numbers can be increased in patients after treatment with antibody-derived agents recruiting NKs as effectors [28,47]. Therefore, after clinical administration of agents related to SPM-2 such as the tandem diabody AFM-13 specific for CD30 and CD16, NKs can expand in human recipients *in vivo*, and sufficient numbers can become available for therapeutic effects, even for malignancies such as Hodgkin Lymphoma that include semi-solid tumor masses [47]. Our results presented above (Figure 5B) suggest that for the patient of the present study, CD3-positive T cells were also greatly reduced in peripheral blood at diagnosis and only recovered together with other leukocytes in the remission phase. Finally, it is not known how many T cells and how many NKs are needed for an effective therapy with such agents. The absolute numbers of NK cells in remission (Figure 4B) may be lower than those of T cells, although absolute numbers have not been determined, but even lower numbers of NKs may be sufficient for therapeutic success.

In the present study, the patient’s NK cells were greatly reduced in relative abundance. At diagnosis their frequency was at least 6–7 fold reduced compared to the two reference samples obtained from the patient in remission and the healthy twin. In the remission sample, normal numbers of NKs with typical cytolytic activity were found. This observation is important for us because SPM-2 was not designed to debulk the mass of leukemic blasts as a frontline agent. Instead, it was designed to act as an adjuvant to further reduce the MRD pool after an initial induction chemotherapy and thus to achieve deeper and longer-lasting remissions.

In this context, the observation that not only the total population of AML blasts from this patient was efficiently lysed by SPM-2 together with MNC effectors (Figure 2B), but that the CD34^pos^ subset was also specifically reduced (Figure 2C), is encouraging. This finding opens the possibility that the AML-LSCs contained within the CD34 compartment were also affected by this treatment. If this were true more generally beyond this single patient, then this would be a welcome result, because AML-LSCs are reported to have increased resistance to treatment with standard chemotherapeutic agents [19,48]. AML-LSCs are typically contained in the CD34^pos^ compartment, and if the LSCs of this patient were similarly sensitive towards cytolysis mediated by SPM-2 in conjunction with NKs as the overall population of CD34^pos^ cells, then this would suggest, that SPM-2 may become a useful new agent for the removal of LSCs. However, these extrapolations are made with due caution, because the AML-LSCs may only account for a minor subset of all CD34^pos^ blasts in this patient and therefore may have escaped lysis in our experiments.

We were further encouraged by our preliminary finding reported here that SPM-2 mediated specific lysis of the CD34^pos^CD38^neg^ CD123^pos^ subset contained within the patient AML blasts, which presumably more narrowly confines the relevant MRD cells than the broader CD34^pos^ compartment. Admittedly, this was only a single initial experiment and further confirmation is needed. Yet, the results obtained so far suggest that SPM-2 in conjunction with functional NKs was capable of effectively eliminating the CD34^pos^CD38^neg^ CD123^pos^ subset, reported by others [24] to include the MRD cells.

No major changes were observed here in the expression profiles of NCRs on NKs between the samples from the patient at diagnosis and in remission, and both profiles were similar to those observed for the healthy sibling. This result was somewhat surprising, because it had been reported by others that differences in NCR expression profiles could be a major cause for impaired functional activity of NKs from AML patients [25-27,29]. One possible explanation for our result is that the expression profile of this particular patient is unique and differs from those of the majority of patients reported elsewhere. Another possible explanation is that in the cases published by others, NCR expression levels were generally compared between NKs from AML patients and those from unrelated healthy donors. This comparison may be less informative than the more rigorous comparison reported here, because it is not excluded that patients in the published studies expressed lower intrinsic NCR levels than the average healthy donor, and that this reduced expression may even have been a cofactor predisposing them to the development of the disease.

## Conclusions

Titers of peripheral blood NKs from a FAB M1 AML patient had recovered to normal levels in remission after induction chemotherapy, and these cells showed normal functional activity in cytolytic assays mediated by the therapeutic triplebody SPM-2. Comparable cytolytic activity of NKs from the patient in remission and a healthy twin were observed both for the patients autologous leukemic blasts as targets, and in benchmark experiments with the standard antibody Rituximab^TM^ against Raji lymphoma cells as targets. The functional impairment of this patient’s NKs obtained at diagnosis probably is not caused by an altered expression pattern of the NCRs, NKp30, NKp40 and NKp46, as suggested in the literature. Expression profiles of these receptors were unaltered in our case. The major cause for a functional impairment of the patients NK cell response at diagnosis appears to be the result of a reduced NK cell titer. Overall, our results indicate that a useful time point to administer SPM-2 for the treatment of AML patients will be the remission phase after induction chemotherapy when blast titers are reduced and titers and functional activity of normal leukocytes have recovered.

## Abbreviations

ADCC: Antibody-dependent cellular cytotoxicity; AML: Acute myeloid leukemia; ELISA: Enzyme-linked immunosorbent assay; FAB: French american british classification of AML; FITC: Fluorescein isothiocyanate; HAM: High-dose cytarabine with mitoxantrone, a standard therapy modality for the treatment of AML [49]; IFN-Gamma: Interferon gamma; MNC: Mononuclear cells; PBMC: Peripheral blood mononuclear cells; RDL: Redirected lysis; TNF-alpha: Tumor necrosis factor alpha; WHO-classification: World Health Organization classification of AML from 2008, as published in 2009 [42].

## Competing interest

The authors declare that they have no competing interests. UJ and GHF are employees of SpectraMab, Munich, Germany.

## Authors’ contributions

TAB participated in the design of the study, performed experiments, analyzed data and helped write the manuscript. SW participated in the design of the study, performed experiments, analyzed data and helped in editing the manuscript. CCR performed experiments, analyzed data and helped in editing the manuscript. IAS contributed to SPM-2 design and performed functional studies. KPH and GHF participated in the design of the study, secured extramural funding and helped in editing the manuscript. FSO managed and coordinated the process of gathering patients, patient material and patient data, helped to secure funding, to plan the study design and to edit the manuscript. All authors read and approved the final manuscript.
